# Genome-wide protein localization prediction strategies for gram negative bacteria

**DOI:** 10.1186/1471-2164-12-S1-S1

**Published:** 2011-06-15

**Authors:** Margaret F Romine

**Affiliations:** 1 Biological Sciences Division, Pacific Northwest National Laboratory, Richland, Washington 99352, USA

## Abstract

**Background:**

Genome-wide prediction of protein subcellular localization is an important type of evidence used for inferring protein function. While a variety of computational tools have been developed for this purpose, errors in the gene models and use of protein sorting signals that are not recognized by the more commonly accepted tools can diminish the accuracy of their output.

**Results:**

As part of an effort to manually curate the annotations of 19 strains of *Shewanella*, numerous insights were gained regarding the use of computational tools and proteomics data to predict protein localization. Identification of the suite of secretion systems present in each strain at the start of the process made it possible to tailor-fit the subsequent localization prediction strategies to each strain for improved accuracy. Comparisons of the computational predictions among orthologous proteins revealed inconsistencies in the computational outputs, which could often be resolved by adjusting the gene models or ortholog group memberships. While proteomic data was useful for verifying start site predictions and post-translational proteolytic cleavage, care was needed to distinguish cellular versus sample processing-mediated cleavage events. Searches for lipoprotein signal peptides revealed that neither TatP nor LipoP are designed for identification of lipoprotein substrates of the twin arginine translocation system and that the +2 rule for lipoprotein sorting does not apply to this Genus. Analysis of the relationships between domain occurrence and protein localization prediction enabled identification of numerous location-informative domains which could then be used to refine or increase confidence in location predictions. This collective knowledge was used to develop a general strategy for predicting protein localization that could be adapted to other organisms.

**Conclusion:**

Improved localization prediction accuracy is not simply a matter of developing better computational algorithms. It also entails gathering key knowledge regarding the host architecture and translocation machinery and associated substrate recognition via experimentation and integration of diverse computational analyses from many proteins and, where possible, that are derived from different species within the same genus.

## Background

Knowledge of the subcellular localization of proteins can provide important insights into protein function and thus is particularly useful in the annotation of genomes and the identification of candidate proteins having functions of interest. For example, microbial proteins that are secreted outside the cell are expected to perform functions associated with cell-cell communication and competition, hydrolysis of membrane impermeable polymers, or creating extracellular structures that enable cell motility, attachment to surfaces, or passage of materials between cells. The discovery of novel surface-localized proteins is useful for the development of drug targets, identification of microbial biomarkers and factors contributing to host invasion, and discovery of more efficient enzymes for use in bioprocesses associated with the breakdown of membrane-impermeable polymers, such as those released during the processing of plant materials for alternative fuel production. In some instances, unexpected localization of proteins belonging to a well studied functional class can lead to exciting new discoveries of cellular function. For example, the discovery that *c*-type cytochromes associated with Mn(IV) and Fe(III) reduction were localized to the cell surface of *Shewanella oneidensis* MR-1 [1] rather than the inner membrane or periplasm where respiratory proteins are typically found, initiated a whole new field of research in extracellular respiratory metabolism.

A wide variety of computational tools have been developed as a rapid, inexpensive means to predict protein localization using only amino acid sequence information. New tools continue to be developed with improved accuracy or specificity making it difficult to decide which one(s) to use for genome-wide prediction of protein locations. The primary improvements to predictive accuracy center on the identification of the substrates of the Sec inner membrane export system, which is responsible for translocation of the majority of extra-cytoplasmic proteins across the cytoplasmic membrane in bacteria [2] and the Tat inner membrane export system which translocates a smaller number of proteins in a pre-folded state [3]. However, bacteria with dual membranes also encode additional machinery for export of proteins from the cytoplasm, for inserting them in the outer membrane, or secreting them beyond the outer membrane. The protein substrates of these systems carry N- or C-terminal signal peptides that are distinct from those recognized by Sec and Tat, or lack them all together, thus requiring the application of alternative computational tools or approaches to identify them. Consequently, prediction of protein localization at the genome scale requires combining multiple tools/methods to account for substrates of both the common export systems, such as Sec, and the less frequently used export or secretion systems.

**Figure 1:**
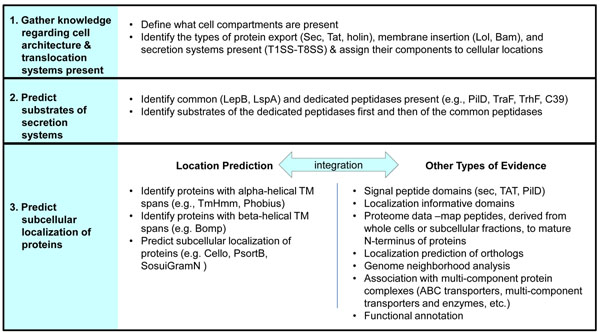
General strategy for predicting protein localization in gram negative bacteria.

In this report, we present lessons learned while curating protein localization predictions in 19 strains belonging to the gram negative Genus *Shewanella* and a generalized workflow (Figure 1) for conducting these analyses that incorporates computational predictions of signal peptide occurrence, subcellular localization, protein domain content, and function with experimental data. The combined genomes of these 19 sequenced strains encode an exceptionally diverse set of secretion systems, including all of named types except the type 4a secretion system (T4aSS), and thus this workflow serves as a useful model for developing strain-specific protein localization prediction workflows in other organisms.

## Results and discussion

### Assessment of the cell architecture

Prior to applying available bioinformatics tools to predict protein localization, it is important to first establish what types of subcellular compartments are present in the organism of interest. The information can then be used to develop a strain-specific strategy for predicting protein localization at the genome scale. Electron microscopy and the genome annotation are useful resources for determining the compartmental organization of the host, but are limited to detection of structures present under the conditions used to generate the sample. However, when supplemented by information garnered from genome annotations this limitation can be overcome. In the sequenced shewanellae, manual curation of the genome annotation suggested that 1) most of the strains harbor at least one bacteriophage within their genomes, some of which have been observed as distinct entities in stressed cultures cells [4,5] and 2) under selected growth conditions *S. benthica* and *S. putrefaciens* strains CN-32 and W3-18-1 [6] will produce cytoplasmic microcompartments that house specific enzymes and associated reactions that benefit from the resulting secluded environment [7]. These observations and sequence-based predictions should be taken into account when predicting protein localization. Bacteriophages encode viral structural proteins that are not components of the cell and, therefore, not appropriate targets for predicting subcellular localization. The genes that encode these structural proteins are frequently co-localized in operons [8] and can often be identified through blast analysis against domains/proteins stored in the Aclame database [9]. Proteins likely to be encapsulated in microcompartments, on the other hand, can be identified by searching for proteins that exist only in organisms encoding microcompartment structural proteins (identified by hits to pfam00936) and frequently are encoded in the same neighborhood with them.

### Identification of protein export and secretion systems

Once the sites that proteins are expected to localize to have been identified, one should proceed with identifying the suite of protein translocation systems that are encoded in the strain(s) of interest. In gram negative organisms, the export of proteins from the cytoplasm to the periplasm is mediated by the inner membrane Sec [10] or Tat [3] translocases while their subsequent insertion in the outer membrane requires the Bam [11] and sometimes Lol [12] systems. Since these systems are broadly conserved their key components can readily be identified by searching for orthologs of their respective universally conserved protein components (Table 1). For strains harboring double-stranded DNA or RNA phage, an additional route across the inner membrane is expected for export of the endolysin that initiates cell lysis. This translocase is encoded by the phage genome, usually adjacent to the endolysin gene, and comprised of a single protein (holin) which is a small inner membrane protein having a C-terminus enriched in basic amino acids [13].

**Table 1:** Core components and associated domains of gram negative export and sorting systems

Export/Insertion System	Function	Core components	Domains	Localization^1^	Comments
General (Sec)	Translocation of unfolded proteins across the inner membrane	SecY	TIGR00967 Pfam00344	IM	Signal peptides cleaved by LspA tend to be shorter than those cleaved by LepB
		SecE	TIGR00964 Pfam00584	IM	
		SecG	TIGR00810 Pfam03840	IM	
Twin arginine (Tat)	Translocation of folded proteins across the inner membrane	TatA	TIGR01411 Pfam02416	IM	respiratory proteins that require cytoplasmic enzymes to covalently attachment metal cofactors (e.g. have iron sulfur, copper, molybdopterin) are expected substrates
		TatB	TIGR01410 Pfam02416	IM	
		TatC	TIGR00945 Pfam00902	IM	
Holin	Translocation of phage endolysin across the inner membrane	Holin	Numerous, but Genus-specific	IM	Encoded near endolysin in double-strand phage
Lol	Insertion of lipoproteins in the outer membrane	LolB	TIGR00548 Pfam03550	LP-OM	beta and gamma Proteobacteria also have LolA having TIGR00547 and pfam03548 domains
		LolC	TIGR00548 Pfam03550	IM	
		LolD	TIGR00221	Cyt-IM assoc	
		LolE	TIGR02212	IM	
Bam	Insertion of beta barrel proteins in the outer membrane	BamA	TIGR03303	OM	With the exception of proteins having large periplasmic domains, expect a genus-specific C-terminal sorting motif
		BamB	TIGR03300	LP-OM	
		BamC	TIGR03302	LP-OM	
		BamD	pfam06804	LP-OM	
		BamE	Pfam04355	LP-OM	

^1^Abbreviations - inner membrane (IM), outer membrane (OM), cytoplasmic, but associated with the inner membrane (Cyt-IM assoc), lipoprotein localized to the outer membrane (LP-OM)

In addition to these export and sorting systems, gram negative bacteria may also encode protein secretion systems, named T1SS-T8SS, that translocate proteins to sites beyond the outer membrane [14]. Secretion systems are often poorly annotated by automated pipelines due to the fact that certain components of different classes of secretion systems (e.g. T2SS and T4SS components) have significant sequence similarity to one another while others, that belong to the same class and that are functionally equivalent, have little similarity to one another (e.g. pilin proteins). In addition, many secretion systems have not yet been characterized and/or informative domains that detect their signature components have yet to been defined and deposited in public databases. Fortunately, the genes encoding the key components of these systems are typically co-localized on the genome and thus one can often use genome context analysis to readily identify their constituents and assign them to appropriate secretion classes.

In *Shewanella*, identification of the outer membrane channel-forming component of these systems (also called secretins or ushers) via domain analysis proved to be an excellent starting point for finding genomic loci that encode secretion systems. Using the 18 domains listed in Table 2, it was possible to identify the genomic loci that encoded 176 putative extracellular secretion systems in 19 sequenced *Shewanella*. Only 95 of these putative secretion systems belonged to the core secretion systems present in every strain, demonstrating the importance of considering these strain-specific differences when predicting protein localization. With the exception of TolC, all of the secretins were encoded in the same genomic loci as the other secretion system components, making it easier to identify and annotate the other genes associated with these apparatus. The lone exception was a subset of the T1SS apparatus, which were predicted to depend on a common TolC secretin that is also responsible for efflux of non-protein substrates, such as drugs and heavy metals. These T1SS were identified by searching for proteins with domains such as TIGR01843 (HlyD family) or TIGR01842, TIGR01846, TIGR03375, and pfam0341 (PrtD, HlyB, and LssB; bacteriocin exporter families) which identify the membrane fusion and permease components of T1SS systems, respectively. In *Shewanella*, each of the loci identified also encoded candidate T1SS substrates, which are typically large proteins lacking a signal peptide with an overall amino acid composition typical of extracellular proteins [15].

**Table 2:** Domains that Identify Secretins and Ushers in *Shewanella*

Domain	Short Model Descriptor	Secretion System	Proteins Detected	Predicted Localization in *Shewanella*
pfam02321	Outer membrane efflux protein	T1SS^1^	AggA	OM
TIGR01844	type I secretion outer membrane protein, TolC family	T1SS	TolC, AggA	OM
TIGR02519	pilus (MSHA type) biogenesis protein MshL	T2bSS	MshL	OM lipoprotein
pfam07655	Secretin N-terminal domain	T2bSS	MshL	OM lipoprotein
TIGR02515	type IV pilus secretin (or competence protein) PilQ	T2bSS	PilQ	OM lipoprotein
pfam00263	Bacterial type II and III secretion system protein	T2a-cSS, T3aSS	GspD, PilQ, MshL, YscC, RcpA, SspD	mixed
pfam03958	Bacterial type II/III secretion system short domain	T2a-bSS, T3aSS	GspD, PilQ, YscC	mixed
pfam02107	Flagellar L-ring protein	T3bSS	FlgH	OM lipoprotein
pfam03524	Conjugal transfer protein	T4bSS	TrbG	OM
pfam03895	YadA-like C-terminal region	T5cSS		OM | extra
pfam03797	Autotransporter beta-domain	T5aSS		OM | extra
pfam06586	TraK protein	T4bSS	TraK, TrhK	OM
pfam07660	Secretin and TonB N terminus short domain	T2bSS^2^	PilQ, MshL	OM lipoprotein
TIGR02516	type III secretion outer membrane pore, YscC/HrcC family	T3aSS	YscC	OM
TIGR02756	type-F conjugative transfer system secretin TraK	T4bSS	TraK	OM
TIGR03352	type VI secretion lipoprotein, VC_A0113 family	T6SS	SciN	OM lipoprotein
pfam00577	Fimbrial Usher protein	T7SS	PapC/FimD	OM
pfam03783	Curli production assembly/transport component CsgG	T8SS	CsgG	OM lipoprotein

^1^ Also detects OM component of drug and metal efflux pumps

^2^ also detects some TonB receptor proteins

While suitable for detecting many of the secretion systems, the domains listed in Table 2 were not able to detect all of the predicted outer membrane protein translocases in the sequenced shewanellae, requiring that other approaches are taken to identify them. For example, protein localization predictions (described below) and comparative genome context analysis can be used to identify commonly occurring genomic loci that encode putative extracellular proteins along with putative outer membrane or lipoproteins. Other types of functional evidence (e.g. domain content, sequence similarity, and literature searches for experimental data on similar proteins) can then be gathered and reviewed for further clues that are indicative of protein secretion machinery. This approach led to the discovery of a conserved five gene locus in two *Shewanella* that includes proteins (previously annotated as hypothetical) with similarity to the recently identified components of the Fap amyloid fiber [16].

T5SS systems, in which the secretin and extracellular function are encoded in the same protein were particularly difficult to confidently identify since the channel forming domain of these systems are highly variable in sequence and currently only detectable by two domains, PF03797 and PF03895 [17]. A review of the literature revealed a new T5SS subclass (T5dSS) that is present in all of the sequenced *Shewanella* and lacks these domains [18], instead having C-terminal domains (PF07244 and PF01103) that are characteristic of the BamA component of the outer membrane protein assembly complex and an N-terminal patatin domain, which is frequently found in extracellular proteins. The orthologous *Shewanella* proteins were all predicted to have a Sec signal peptide by SignalP and to reside in the outer membrane by Bomp, but predicted to localize extracellularly by PsortB and Subloc or to a mixture of outer membrane and extracellular environment by Cello and SosuiGramN. Phobius also detected a signal peptide, but suggested that a single transmembrane span remains at the C-terminus. This region matches the TIGR03501 gamma proteobacterial enzyme C-terminal transmembrane domain, an extracellular location informative domain that is predicted to be proteolytically removed prior to protein secretion (Dan Haft, personal communication). These observations suggest that additional novel T5SS can potentially be identified by searching for proteins with similar mixed evidence of location. Another feature to look out for is the occurrence of exceptionally long Sec leaders known to occur in some T5SS proteins [19,20]. Since its length may preclude its detection by computational tools designed to detect signal peptides (see below), manual inspection of candidate dual domain T5SS translocases for Sec leaders may be necessary.

### Detection of signal peptidases and signal peptides

**Figure 2:**
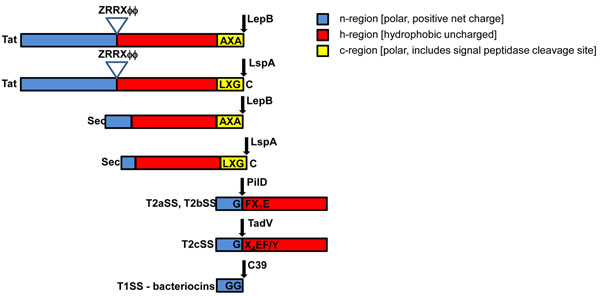
N-terminal signal peptides detected in *Shewanella*. Note that some T5SS substrates have been reported to possess an additional n- and h- region at the N-terminus and thus the position of the signal peptidases cleavage site would likely go undetected by standard predictors such as SignalP. Arrows indicate the position at which the signal peptide is cleaved by the respective signal peptidases. Conserved sequences are indicated, with X denoting any amino acid. The twin arginine motif is denoted by ZXRRXϕϕ, where Z=hydrophilic residue and ϕ=hydrophobic residue.

Once the suite of export and secretion systems present in the strain of interest are identified, a review of the recent literature is warranted to determine whether conserved sequence features are expected in their substrates. Most protein localization systems recognize conserved motifs encoded at either the N- or C-terminus of their substrates (Figure 2). Substrates of Sec, the predominant pathway for inner membrane protein translocation, are received in their unfolded conformation and have a characteristic N-terminal signal peptide [21] that is removed after export by either signal peptidase I (LepB) [22] or, in the case of lipoprotein substrates, by signal peptidase II (LspA) [23]. Popular localization predictors, such as SignalP [24-26] and PsortB [27], are designed to detect only LepB processed Sec substrates. Therefore, tools such as LipoP [28] or Lipo [29] must be used to identify lipoprotein substrates of the Sec translocator. TatP [30] was developed to identify signal peptides present in substrates of the Tat exporter, which is responsible for translocation of folded proteins, many of which bind redox cofactors [31]. However, this tool is unable to detect lipoprotein substrates, as it has only recently been recognized they could be substrates of this system. *Shewanella* sp. are known to use Tat to translocate the molybdopterin-binding lipoprotein subunits of the DMSO [32] and arsenate [33] reductases across the inner membrane and hence we expected to find characteristic Tat signal peptides with LspA cleavage sites when analyzing their genomes. Since LipoP is unable to detect Tat signal peptides, Tatfind [34] was used to identify proteins having them and then manually searched for an adjacent LspA cleavage site. A recent genomic survey using an algorithm based on the DOLOP database of lipoproteins [35] and TatP rules, suggested that lipoproteins are Tat substrates in numerous other organisms as well [36]. It should also be noted that proteins which form complexes with Tat substrates can be exported by Tat even though they lack a Tat signal peptide. This phenomenon has been demonstrated for translocation of multi-subunit enzymes such as hydrogenase [37]. Since hitchhikers are not detected by TatP or Tatfind, they need to be manually identified through searches for proteins that are encoded in the neighborhood of TAT substrates and having amino acid composition characteristic of extra-cytoplasmic proteins and/or functional annotations suggesting participation in multi-subunit enzymes.

The occurrence of alternative signal peptidases are expected in organisms that possess type II or IV secretion systems since they are necessary for maturation of the pilin/pseudopilin components of these systems. T2SS and T4SS peptidases can be detected by searching for proteins belonging to Merops [38] families A24A and S26, respectively (Table 3). In *Shewanella* a single peptidase, PilD, processes the pilin components of all three subclasses of T2SS and most of its substrates are identifiable by matches to pfam07963 and/or TIGR02532, while others (e.g., GspK, PilX, PilW) that have an imperfect match to the PilD cleavage site could be detected only by similarity at the N-terminus of the mature protein to other PilD substrates or matches to pfam03934 (GspK). Pili associated with IncJ and IncP conjugative systems were detected by matches to TIGR02758 and pfam04956, respectively, while IncH and IncF pili were recognizable only by homology to previously characterized pili associated with these systems. Class I and II bacteriocins are processed at the N-terminus by a C39 family peptidase whose activity is encoded in the N-terminus of the permease component of the T1SS system responsible for bacteriocin secretion. These small proteins are often missed during automated annotation, but can typically be found in genomic loci encoding this characteristic T1SS by searching for nearby small open reading frames that encode proteins with a characteristic twin glycine signal peptide [39].

**Table 3:** Characteristics of signal peptidases and target signal peptides

Model Signal Peptidase	Example Protein	Merops Family & domains	Translocation system	Signal peptide domain	Example substrates	Domains	Comments
N-terminal Processing							

PilD PulO GspO	SO_0414	A24A	T2aSS	pfam07963	GspGHIJK	pfam02501 pfam03934 pfam08334 pfam12019	Signal peptides similar, except ones in IVb pili are longer (~25 aa) than others (~7 aa), GspK, PilX, PilW are not detected by pfam07963
				
			T2bSS		type IVa pili		
		
					type IVb pili		

TadV	Spea_2010	A24A	T2cSS		Flp, TadEF		

LspA	SO_3531	A08 pfam01252	Sec		Lipo-proteins		
			
			Tat	TIGR01409 pfam10518			

NA^1^	SO_A0049	C39	T1SS	TIGR01847	class Ia & IIa-b bacteriocin, microcins	pfam01721 pfam10439 pfam10439	The signal peptidase activity is encoded in the permease component of the T1SS system that exports the bacteriocin

LepB	SO_1347	S26A pfam00717	Sec		exported proteins		
			
			Tat	TIGR01409 pfam10518			

C-terminal processing							

?			T4bSS - IncF		TraA		mature TraA is about ~68 aa in length with two TM spans that circularizes

TrhF	Shewana3_4209	S26A	T4bSS - IncH		TrhA HdtZ		Substrates have Sec signal peptide that is cleaved by LepB
	
TraF	Sputw3181_1142	S26A	T4bSS - IncJ		TraA	TIGR02758	
	
TraF	Shewana3_1267	S26C pfam00717	T4bSS - IncP		TrbC	pfam04956	

### Gene model improvement

Because the detection of signal peptides is an important step in localization prediction, errors in prediction of the 5’ end of a gene can displace or truncate N-terminal signal peptides and thus impact the accuracy of localization predictions. Significant improvements have been made in the ORF calling algorithms since the advent of whole genome sequencing and, therefore, the gene models for genomes produced with the earlier generation ORF calling algorithms can be readily improved by comparing the output of the newer algorithms to those used in the original Genbank deposit, or simply using the newer gene model predictions. The output of several of these newer algorithms (Glimmer v. 3, Prodigal v. 2, GeneMarkHMM-2.6r, and GeneMark-2.5m) are pre-computed and available to the research community via FTP from NCBI Refseq (ftp://ftp.ncbi.nih.gov/genomes/Bacteria).

Another means to improve the gene model is to map the termini of transposons, insertion sequences, and other mobile elements in the genome as we reported previously for *S. oneidensis* MR-1 [40]. This task is not routinely part of the automated genome annotation process and results can reveal that seemingly intact genes are truncated at their 5’ end or interrupted and hence localization predictions can be erroneous. Identification of mobile elements is facilitated by the use of resources like ISfinder [41] and ACLAME [9] that provide information regarding the sites targeted by and characteristics of the termini of insertion elements and prophage, respectively. Programmed recoding of genes, whereby genes are translated by non-standard rules (e.g., programmed ribosomal frameshifting, translational bypassing, and utilization of alternative tRNAs to decode stop codons as an amino acid) can also be missed during automated annotations, sometimes even resulting in their erroneous annotation as pseudogenes. The Recode database (http://recode.ucc.ie/) [42] has compiled numerous examples of recoded genes and thus provides a useful resource for identifying genes likely to be subject to recoding.

Comparative analysis of the protein size, domain content, and localization predictions among orthologous proteins can also prove useful for identifying errors in gene models. Inconsistencies in these values among orthologous *Shewanella* proteins could often be eliminated by adjusting gene start/stop positions or membership within a predicted orthologous group. In some cases, inconsistencies suggested that one or more members of the group possessed longer signal peptides than detectable by programs such as SignalP or LipoP or that a proposed signal peptide was more likely an uncleaved N-terminal transmembrane domain. As mentioned earlier, unusually long leaders would be expected in some T5SS autotransporters and the secreted component of T5SS two partner secretion systems since some members of this class have signal peptides that are preceded by an additional charged (n-region) and hydrophobic domain (h-region) [20].

Proteomic data can prove especially useful for improving the gene model, but there are several caveats to their use in validation of genes models that one should be aware of. Trypsin, which specifically cleaves proteins C-terminal to arginine (R) or lysine (K) residues, is the most common enzyme used to digest proteins into fragments of suitable number, size, and charge for subsequent sequence identification by gel-free mass spectrometric-based methods for global characterization of proteins. The C-terminus of each peptide generated is expected to be an R or K and the N-terminus should map adjacent to an R or K in the parent protein. In theory, the only peptides with ends that do not match these criteria, should result from host-mediated proteolytical processing (e.g. by LepB) of the parent protein prior to its tryptic digestion and thus detection of partially tryptic peptides should be indicative of host-mediated post-translational processing of proteins or incorrect assignment of a start codon. However, in practice, partially tryptic peptides can also result from the harsh conditions associated with sample processing, sample fragmentation during ionization, or erroneous peptide identification [43-45]. Therefore, when using proteome data for identifying the N-terminus of mature proteins it is prudent to consider only partially tryptic peptides that, among all peptides detected, are the ones mapping most closely to the N-terminus of the parent protein. Furthermore, the N-termini of these peptides should map to a site that is consistent with predicted protease cleavage sites. In *Shewanella*, the most frequently encountered proteolytic processing event detected in *Shewanella* was due to cleavage by AmpP or Map (both present in all the *Shewanella* genomes), which remove the N-terminal methionine when it is adjacent to Pro or a small amino acid (Ala, Ser, Gly, Cys, Thr, Pro, or Val), respectively [46,47]. In most cases where a partially tryptic peptide did not map to position 2 of the parent protein (AmpP or Map processed) the detected partially tryptic peptides mapped to signal cleavage sites predicted by SignalP or TatP. A notable exception was the long signal peptide (68 amino acids) found in the small subunit of the NiFe hydrogenase, an expected TAT substrate whose cleavage was not recognized by TATP (except in 1 out of 17 strains having this protein) but for which validating partially tryptic peptides were detected in 4 different strains of *Shewanella* (see Additional file 1) (M. Romine, unpublished results).

Global analyses of cellular proteomes by mass spectrometry uses the protein sequences deduced from the genomic sequence for peptide matching and thus peptides that map outside of the defined gene termini go undetected. Therefore, searches of MS-MS spectra against protein sequences derived from translations between all stop codons (stop-to-stop databases) or between each stop codon and the furthest upstream start codon (start-to-stop databases) have also been used to increase the number of identifiable peptides in hopes of validating earlier start sites or missed open reading frames [48]. However, non-standard start codons, such as GTG and TTG, are frequently used in bacteria and archaea, but would not be translated as methionine in stop-to-stop *in-silico* translations. Therefore, N-terminal peptides produced from proteins whose translation is initiated at alternative start codons would still go undetected and consequently the returns from such an effort are diminished. Furthermore, since these databases are significantly larger, the chance of erroneous peptide matching is significantly increased and thus warrants manually evaluating each peptide mapping outside pre-defined open reading frame, especially when the peptide is infrequently detected in samples analyzed.

### Predicting protein localization

A variety of different computational tools are available for predicting subcellular location, but only a few enable batch analysis via a web interface and each has certain limitations. A comparison of subcellular localization or signal peptide predictions produced with popular computational tools having batch analysis available (Table 4) revealed frequent inconsistencies in location prediction or signal peptide detection among members of the same ortholog group, even after adjusting gene models or ortholog group membership. Disagreements in predictions for a single protein were also common among the predictions generated by different tools designed for the same purpose. The extent of the problem is shown in Tables 5 and 6, which compare results of different analyses among each set of 19 proteins belonging to one of the 1990 core ortholog groups in *Shewanella*. At best, only 70% of the groups had consistent subcellular localization prediction suggested for all its members. A comparison of the predictions produced by PsortB, Cell, and SosuiGramN for all 81,619 predicted proteins revealed that just under half of them (39,538) were consistent in localization prediction. The disagreements generally reflected that some tools are better suited for certain types of predictions (e.g. 75 of the predicted SignalP false positives were due to incorrect classification of proteins having signal peptides cleaved by PilD or LspA) while inconsistencies in predictions among orthologs simply revealed the uncertainly of these predictions.

**Table 4:** Computational Tools used in Studies of *Shewanella*

Name	URL	Use	Limitations
LipoP	http://www.cbs.dtu.dk/services/LipoP/	primarily prediction of Sec signal peptides that are cleaved by LspA but also provides prediction of inner membrane or cytoplasmic localization as well as LepB cleavage	does not detect Tat substrates
Lipo	http://services.cbu.uib.no/tools/lipo	prediction of Sec signal peptides that are cleaved by LspA	does not detect Tat substrates
SignalP	http://www.cbs.dtu.dk/services/SignalP/	prediction of Sec signal peptides that are cleaved by LepB	does not detect Tat substrates
Phobius	http://phobius.sbc.su.se/	prediction of alpha helices in inner membrane proteins, distinguishing N-terminal TM from signal peptides	
TmHmm	http://www.cbs.dtu.dk/services/TMHMM/	prediction of alpha helices in inner membrane proteins	Signal peptides are often erroneously counted as TM spans
Bomp	http://services.cbu.uib.no/tools/bomp	prediction of beta barrel spans in outer membrane proteins	
Cello	http://cello.life.nctu.edu.tw/	prediction of localization (Cyt, IM, Peri, OM, Extra)	does not predict lipoprotein location in OM or IM
Sosui-GramN	http://bp.nuap.nagoya-u.ac.jp/sosui/sosuigramn/sosuigramn_submit.html	prediction of localization (Cyt, IM, Peri, OM, Extra) in gram negatives only	does not predict lipoprotein location in OM or IM, no scores given
Subloc	http://www.bioinfo.tsinghua.edu.cn/SubLoc/	prediction of localization (Cyt, Peri, Extra)	not appropriate for membrane bound proteins
PsortB	http://www.psort.org/psortb/index.html	prediction of localization (Cyt, IM, Peri, OM, Extra)	does not predict lipoprotein location in OM or IM, many proteins assigned
TatP	http://www.cbs.dtu.dk/services/TatP-1.0/	prediction of Tat and Sec signal peptides	does not detect lipoproteins that have Tat signal peptide; some very long signal peptides not detected
Tatfind	obtained from Dr. Pohlschroder ^1^	Prediction of Tat signal peptides	does not require the presence of an adjacent LepB or LspA site or that it occurs at the protein N-terminus (though this can be advantageous when the start codon prediction is wrong)

^1^ For correspondence. E-mail pohlschr@ sas.upenn.edu

**Table 5:** Tool Performance Across 19 Proteins in Each of the 1990 Core Ortholog Groups

Test	Tool	Groups with no match	Disagree with Curation	Groups with match	Disagree with Curation	Groups with mixed predictions	Curated as having Match
Sig Pep cleaved by LspA	LipoP 1.0	1911	0	49	0	30	61

Sig Pep cleaved by LepB	SignalP-NN 3.0	1482	4	158	39	350	169
	
Sig Pep cleaved by LepB	SignalP-Hmm 3.0	1447	1	247	89	296	

Sig Pep recognized by TAT	TatP 1.0	1962	0	5	2	23	5

Inner membrane protein	TmHmm 2.0	1417 (1)^1^	1	390 (103)	21	183 (29)	403 (133)
	
Inner membrane protein	Phobius	1505 (14)	14	349 (72)	7	136 (47)	

Outer membrane protein	Bomp	1934	11	13	2*	43	32

^1^Values in parentheses indicate number of proteins predicted to have only 1 transmembrane span.

**Table 6:** Performance of Localization Predictors Across 19 Proteins in Each of the 1990 Core Ortholog Groups

Subcellular localization	Curated Localization^1^	Cello 2.5^2^	SosiuGn	Subloc	PsortB 3.02
extracellular	40	7 (7)^3^	7 (7)	24 (2)	14 (11)
Outer Membrane	32	16 (12)	21 (12)	NA	20 (14)
Periplasm	176	25 (16)	20 (18)	72 (24)	21 (20)
Inner Membrane	403	222 (222)	281 (278)	NA	349 (294)
Cytoplasm	1339	750 (737)	780 (779)	1277 (977)	976 (970)
Total	1990	1020	1109	1373	1380

^1^Lipoproteins localizing to the outer or inner membrane were counted as periplasmic, while those predicted to localize to the cell surface were counted as extracellular. T5SS autotransporters were counted as extracellular.

^2^Only Cello values for which a single location was predicted are included in these counts.

^3^Numbers in parentheses indicate the number of groups that are in agreement with curated locations.

**Figure 3:**
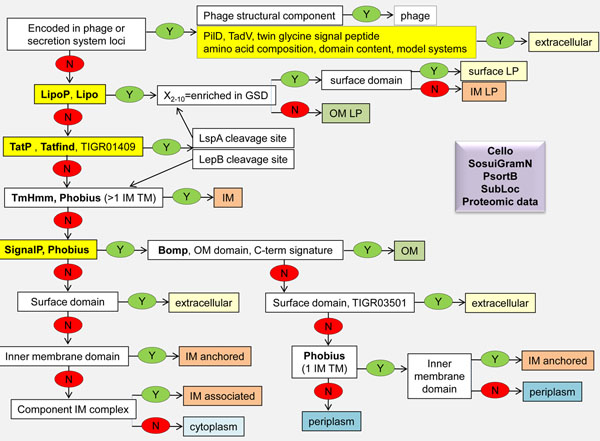
Decision tree for predicting localization of proteins in gram negative bacteria.

To address these issues, a decision tree (Figure 3) was developed as a guide for using predictions of the occurrence of sorting signals or location informative domains to support or refute global subcellular location predictions or proteomics data from subcellular fractions. While majority voting could often be used to predict location, significant manual curation of gene models (4,208 proteins so far), ortholog grouping, and location assignments was necessary to resolve conflicts in location evidence gathered. It should be noted that the tools listed in the decision tree simply reflect those that were used in analysis of *Shewanella*. Those chosen were limited to ones that allow batch analysis on-line and that are more broadly used by other researchers, but are not necessarily the most accurate ones currently available. The tools listed can be replaced or supplemented by other tools (recently reviewed in [49,50]) that are better suited to the organism of interest or yield improved accuracy, recognizing that some may require local installation of software to make genome-scale analyses feasible.

The prediction schema is initiated with the curation of secretion systems, whose components often have distinct signal peptides that are not recognized by predictors listed or that are secreted during assembly of the machinery. In addition, the structural components of bacteriophage are identified at this stage as they would otherwise often be erroneously predicted to localize to the cell envelope. Next automated searches for signal peptides are conducted, working first on the less common signal peptides associated with lipoproteins and Tat substrates and then followed by searching for transmembrane spans and Sec signal peptides. A comparison of the latter two results assisted in distinguishing signal peptides from transmembrane spans, but the availability of additional information (e.g., expected location of a protein based on annotation, detection of peptides that map at or near the N-terminus) was generally needed for deciding whether the N-terminus was removed versus being retained for anchoring a protein in the membrane. Domain content and functional annotations were used through-out this decision tree to increase the confidence and accuracy of the predictions. Location informative domains were identified by searching for Pfam and TIGRfam domains that consistently occurred only in proteins predicted to localize to the same site and/or had a known association with proteins found in specific subcellular or extracellular compartments. In addition, results of searches for a C-terminal outer membrane localization signature were used to enhance outer membrane location predictions, recognizing that those having large periplasmic domains (e.g. TolC family proteins) are expected to lack these signatures or contain them at internal sites instead. This species-specific C-terminal signature consists of alternating hydrophobic residues at positions 5, 7, and 9 from the C-terminus and a Phe or Tyr at the terminus [51,52]. Since shewanellae have numerous TonB receptors (620 in 19 genomes) we used their C-termini to develop a *Shewanella*-specific signature that could be used to search for additional substrates of this system.

Characterization of the protein content of subcellular fractions by mass spectrometry is also a useful type of evidence for assessing protein localization. This information is particularly useful for identifying proteins that are tethered to the membrane via protein-protein or protein-lipid interactions or for condition-specific changes in protein localization which cannot be revealed by analyses of protein sequence content alone. However, results must be interpreted with caution as there can be significant cross-contamination between subcellular fractions which may vary depending on the protocol used to fractionate and analyze the protein content or the cell type being studied. In reviewing the data from LC/MS-MS analysis of *S. oneidensis* MR-1 subcellular fractions prepared with a sarkosyl-based method, we found that fractions with the greatest abundance of peptides partitioned were usually consistent with the predicted locations of the parent protein with the notable exception that many more lipoproteins partitioned to the inner membrane than expected [53]. Sarkosyl was chosen over other detergents because of it compatibility with high through-put MS-based proteomic analysis and reduced time and labor required to conduct the cellular fractionation. While this detergent has been shown to preferentially solubilize inner membrane proteins [54] thus allowing efficient separation of inner and outer membranes, it is possible that it also solubilizes the loosely associated outer membrane lipoproteins.

Alternatively, the predicted localization of these proteins is incorrect. The rules for predicting lipoprotein sorting are based on extensive research on *Escherichia coli* lipoproteins and suggest that lipoproteins with an aspartic acid (D) at position +2 (D^+2^) of the mature protein are retained in the inner membrane while the remainder are attached to the outer membrane by Lol [55]. However, numerous exceptions have been found in other organisms [56-58] suggesting that these rules likely only apply to enterobacteria. Indeed, our analysis of over 3000 predicted lipoproteins in this Genus revealed a lack of consistency in occurrence of D^+2^ in orthologs and that only 5 out of 112 efflux pump membrane fusion lipoproteins, which are expected to be anchored to the inner membrane, have D^+2^. Furthermore, like selected other bacteria [59-62], *Shewanella* can also localize lipoproteins to the outer face of the outer membrane and thus must use alternative sorting signals. While it is known that the T2aSS machinery is responsible for their surface translocation in *Shewanella*[32,63,64], the characteristics of the sorting signals used are currently unknown. The large number of putative lipoproteins identified in this genus and combined knowledge available regarding their localization (experimentally validated as well as predicted based on function or domain content), however, provided a more sensitive means to search for conserved sequences that are characteristic of surface lipoproteins. In *Shewanella* such analyses suggest that enrichment in glycine and serine residues coincides with predicted surface localization (Romine, unpublished results). These same amino acids have recently been reported to be enriched in extracellular proteins [65] and are commonly found in other sorting signals used for secretion of proteins [66,67].

## Conclusions

While the methodological process described here was derived from studies of a Genus that shares many structural and functional features with organisms from which much of our current understanding of translocation models have been developed, the overall strategy described for predicting protein localization should prove useful for studying other microbes as well. Knowledge gathered regarding distinctive architectural features or unusual translocation machinery content (e.g. missing components, duplications) prior to applying automated sequence analysis methods can significantly impact the choice of computational tools to use and subsequent interpretation of the results. Proteomic analyses can be especially useful for confirming predictions or discovering novel sorting signals, while less costly computational localization predictions, conducted at the genome scale, can reveal novel characteristics of an organism that might not be readily derived from functional annotations derived solely from sequence similarity.

## Additional information

Subcellular localization and ortholog grouping predictions (Additional file 2) and associated protein sequences (Additional file 3) that were used to for making calculations provided in tables 5 and 6 are provided in the supplementary material so that interested parties can use them for evaluating their own prediction strategies to those used by the author. However, it should be noted that updates to the gene models and ortholog membership is an ongoing process, with the most current versions available at http://shewanella-knowledgebase.org:8080/Shewanella/. Updated localization predictions are available through the author.

## Competing interests

The author declares that they have no competing interests.

## Supplementary Material

Additional file 1 **Alignment of N-termini of the periplasmic [Ni-Fe] hydrogenase large subunit, HyaB.** Representative N-terminal amino acid sequences from 14 different *Shewanella* species are shown adjacent to their corresponding locus tag. Residues found in the conserved TAT motif are shown in bold. The predicted N-termini of the mature proteins are underscored. The sequences corresponding the most N-terminal peptide identified in four of these microbes [68] using the AMT approach [69] is shown in bold and underscored. Click here for file

Additional file 2 **Curated ortholog grouping and location predictions for 81169 proteins predicted to be encoded in the genomes of 19 *Shewanella*.** Pseudogenes are denoted with an asterisk in the locus tag column. Click here for file

Additional file 3 **FastA file of proteins predicted to be encoded in the genomes of 19 *Shewanella*.** This file includes translations of pseudogenes, with internal stop codons assigned the value ‘X’. Click here for file
