# Supplementary material for: Genome-wide protein localization prediction strategies for gram negative bacteria
Source: BMC Genomics. 2011 Jun 15;12(Suppl 1):S1. doi: 10.1186/1471-2164-12-S1-S1 (PMC3223724; doi:10.1186/1471-2164-12-S1-S1)
Supplement: Additional file 1 — Alignment of N-termini of the periplasmic [Ni-Fe] hydrogenase large subunit, HyaB. Representative N-terminal amino acid sequences from 14 different Shewanella species are shown adjacent to their corresponding locus tag. Residues found in the conserved TAT motif are shown in bold. The predicted N-termini of the mature proteins are underscored. The sequences corresponding the most N-terminal peptide identified in four of these microbes [68] using the AMT approach [69] is shown in bold and underscored. [file 1471-2164-12-S1-S1-S1.docx]

KT99_11163 MDTHAALYRQGQARLDMLRQLNSRDGQSLTSKMKQHDIS**RRDFIK**WSASVTAMLALPLPF

Ssed_1908 MDTHAALYRQGQARLDMLRQLKSRDGQSLTSKMKEHGIT**RRDFMK**WSASVTAMLALPLPF

Sfri_2103 MDTHAALYKQGQDRLDYLRQFAQRQPVSLQQKMANIGIS**RRDFIK**WTASVTAMFALPLPF

Shew_1765 MDTHAALYQQGQARLEELRRLAPRHAQTLQDKMEEHGIT**RRDFMK**WSAAVTAMLALPLPF

Sama_1679 MDTHAALYEQGKQRLETLRSLPRRHEETIFDKMAQHGIT**RRDFMK**WSASVTAMLALPLPF

sputcn32_2088 MDTHAALYEQGKARLEALRQLAPRQQQSLVEKMQQHGIS**RRDFMK**WSAMVTGMLALPLPF

sputw3181_1924 MDTHAALYEQGKARLEALRQLAPRQQQSLVEKMQQHGIS**RRDFMK**WSAMVTGMLALPLPF

Sbal223_2404 MDTHAALYEQGKARLDALRQFAPRQQQSLIEKMQQHGIS**RRDFMK**WSAMVTSMLALPLPF

SO_2099 MDTHAALYEQGKARLDALRQFAPRQQQTLLEKLQQHGIS**RRDFMK**WSAMVTGMLALPLPF

Shewana3_1880 MDTHAALYEQGKARLDALRQLAPRQQQTLIEKLQQHGIT**RRDFMK**WSAMVTGMLALPLPF

Shewmr4_1822 MDTHAALYEQGKARLDALRQFAPRQQQTLTEKLQQHGIT**RRDFMK**WSAMVTGMLALPLPF

Shewmr 7_2155 MDTHAALYEQGKARLDALRQFAPRQQQTLTEKLQQHGIT**RRDFMK**WSAMVTGMLALPLPF

shal_2261 METHEALYQQGLARIEQLRKQKPRHSETLQQKLEQNGIT**RRDFMK**WSASVTAMLALPLPF

Spea_2033 METHEALYQQGVARMEQLRKLQPRTGETLQQKMEQNGIT**RRDFMK**WSAAVTAMLALPLPF

swp_2639 MDTHEALYQQGVARMEQLRQLQPRHSESLKDKMLQNGIS**RRDFMK**WSASVTAMLALPLPF

*:** *** ** *:: ** * :: .*: : .*:****:**:* **.*:******

KT99_11163 STLVAQAAELADRVPLIWLHMAECTGCSESLIRTDTPNLDSLIFDHISLEYHETLMAASG

Ssed_1908 STLVAEAAELADRVPLIWLHMAECTGCSESLIRTDTPNLDSLIFDHVSLEYHETLMAAAG

Sfri_2103 SNLVAEA**AELADRVPLIWLHLAECTGCSESLIR**TDSPNLDTLIFDHISLEYHETLMAASG

Shew_1765 STLVAEAAELADRVPLIWLHMAECTGCSESLVRADTPNLDSLIFDHVSLEYHETLMAAAG

Sama_1679 STLVAEAAELADRVPLIWLHMAECTGCSESLVRADTPNLDSLIFDHISLEYHETLMAAAG

sputcn32_2088 SNLVAEA**AELADRVPLIWLHMAECTGCSESLVR**ADTPNLDSLIFDHISLEYHETLMAASG

sputw3181_1924 SNLVAEA**AELADRVPLIWLHMAECTGCSESLVR**ADTPNLDSLIFDHISLEYHETLMAASG

Sbal223_2404 SNLVAEAAELADRVPLIWLHMAECTGCSESLIRTDTPNLDSLIFDHISLEYHETLMAAAG

SO_2099 SNLVAEA**AELADRVPLIWLHMAECTGCSESLVR**ADTPNLDSLIFDHISLEYHETLMAAAG

ANA3_1880 SNLVAEAAELADRVPLIWLHMAECTGCSESLVRADTPNLDSLIFDHISLEYHETLMAAAG

MR4_1822 SNLVAEAAELADRVPLIWLHMAECTGCSESLVRADTPNLDSLIFDHISLEYHETLMAAAG

MR7_2155 SNLVAEAAELADRVPLIWLHMAECTGCSESLVRADTPNLDSLIFDHISLEYHETLMAAAG

shal_2261 STLVAEAAELADRVPLVWLHMAECTGCTESLIRTTTPNISTLIFDYVSLEYQETLMAAAG

Spea_2033 STLVAEAAELADRVPLVWLHMAECTGCSESLIRTDTPNLDTLIFNHVSLEYHETLMAAAG

swp_2639 STLVAEAAELANRVPLIWLHMAECTGCSESLIRTDTPNLDSLIFDHVSLEYHETLMAAAG

*.***:**********:***:******:***:*: :**:.:***:::****:******:*
